# Effect of attending pregnant women’s conference on institutional delivery, Northwest Ethiopia: comparative cross-sectional study

**DOI:** 10.1186/s12884-019-2537-7

**Published:** 2019-10-12

**Authors:** Melash Belachew Asresie, Gizachew Worku Dagnew

**Affiliations:** Department of Reproductive Health and Population Studies, School of Public Health, College of Medicine and Health Sciences, Bahir Dar University, Bahir Dar, Ethiopia

**Keywords:** Pregnant women’s conference, Institutional delivery, Northwest Ethiopia

## Abstract

**Background:**

Institutional delivery is the cornerstone reducing maternal mortality. Community-based behavioral change interventions are increasing institutional delivery in developing countries. Yet, there is a dearth of information on the effect of attending pregnant women’s conferences in improving institutional delivery in Ethiopian. Therefore, this study was aimed to assess the effect of attending pregnant women’s conference on institutional delivery, Northwest Ethiopia.

**Methods:**

Community-based comparative cross-sectional study was conducted in 2017 among 871 women who gave birth within the last 12 months (435: pregnant women’s conference attendants and 436: pregnant women’s conference non-attendants). Participants were selected by using a multistage-simple random sampling technique and a structured interviewer-administered questionnaire was used for data collection. Both descriptive and logistic regression analyses were performed using SPSS V.23. A *P*-value less than or equal to 0.05 at 95% confidence interval was set to test statistical significance.

**Results:**

Institutional delivery among women who attended pregnant women’s conferences was 54.3%, higher compared with 39.9% of women who didn’t attend the conference. Likewise, the level of well-preparedness for birth was higher among women who attended the conference (38.9%) compared with their counterparts (25.7%). Being knowledgeable on childbirth (AOR = 1.7, 95%CI: 1.2, 2.8) and postpartum danger signs (AOR = 14.0, 95%CI: 4.6, 40.0), and discussed with partners/families about the place of birth (AOR = 7.7, 95%CI: 3.6, 16.4) were more likely to institutional delivery among women who attended pregnant women’s conference. Whereas, among women who didn’t attend the pregnant women’s conference, being knowledgeable about pregnancy danger signs (AOR = 3.6, 95%CI: 1.6, 8.1) were more likely to institutional delivery. In addition, the nearest health facility within 1 h of walking and well-preparedness for birth and its complication were found positively associated with institutional delivery in both groups.

**Conclusion:**

Institutional delivery was low in both groups compared to the national plan, but was higher among women who attended the conference. Similarly, women’s knowledge of obstetric danger signs and preparation for birth and its complication was higher among women who attended the conference. Therefore, encouraging women to attend the pregnant women’s conference and discuss with their families about the place of delivery should be strengthened.

## Background

Globally, maternal mortality had reduced by 44% in the time between 1990 and 2015. More than 35 women died worldwide every hour due to the complications of pregnancy and childbirth in 2015, of which, Sub-Saharan Africa alone accounts for almost two-thirds (66%) of global maternal death [1, 2]. In Ethiopia maternal death is the highest in the world which accounts, 412 deaths per 100,000 live births in 2016 [3]. Sustainable development goal has planned to reduce the maternal mortality ratio (MMR) from 216 to 70 per 100,000 live births by 2030 [4].

Institutional delivery is the cornerstone reducing maternal Mortality. However, institutional delivery use is low [3, 4]. Behavioral changing community-based intervention is globally endorsed to reduce delays for care by increasing obstetric danger signs and birth preparedness knowledge [5, 6]. Developing countries have recently worked in behavior change and community mobilization interventions to increase institutional delivery [7–9].

The government of Ethiopia has strived to improve institutional delivery by introducing innovative practices like community participation, the adaptation of cultural practices at health facility level such as preparation of “maternal waiting home” at the health facility, making the service free of charge and provision of the ambulance for transportation of pregnant women during labor and delivery. Health developmental army and health extension workers having great changes in the utilization of maternal and childcare [10–13]. In addition to this, Ethiopian minister of health launched community-based intervention so-called “pregnant women’s conference” (PWC) to enhance obstetric danger sign awareness and institutional skilled maternal health service utilization. PWC is aimed to increase institutional delivery, by reducing delay in deciding to seek care and in the process of seeking care through enhancing the knowledge of obstetric danger signs and encourage proactive preparation toward childbirth and against the occurrence of any complication ahead of time among pregnant women. Every pregnant woman expected to attend at least three times during each pregnancy. It is given by Nurse/Midwives monthly at their kebele level/the smallest administrative unit in Ethiopia/ and coordinated by health extension workers. There are studies in Ethiopia about institutional delivery but evidence on the effect of PWC on institutional delivery is scant. Therefore, this study was aimed to assess the effect of attending a pregnant women’s conference on institutional delivery by comparing women who attended the conference and women who didn’t attend the conference during their last pregnancy in rural Northwest Ethiopia.

## Methods

### Study area and period

The study was conducted in rural Libo Kemkem District Northwest Ethiopia from February 15 to March 26, 2017. Libo Kemkem District is found 645 km away from Addis Ababa’s capital city of Ethiopia. The district has 29 rural Kebeles & 5 urban Kebeles with a total population, 261, 170 in 2016/17 according to the population projection made from the 2007 Ethiopian population and housing census, of which, the rural population accounts 223,378 people [14].

### Study design

Community-based comparative cross-sectional study design was conducted among women who gave birth within the last 12 months.

### Sample size

The minimum sample size for each group was calculated using the formula of two-sample comparisons of proportion by using Epi-Info V.7 StatCalc, cohort or cross-sectional, based on the following assumptions: confidence level 95%, power 80, and the proportion of institutional delivery among intervention and controlled group from the Burkina Faso study was 56 and 36% respectively, as there is no similar study conducted in the country to be used as a base to determine the sample size [15]. Adding of 5% non-response rate and multiplied by 2 (design effect because to be used multistage sampling method), the final minimum sample size calculated for each group was 450.

### Study population and sampling technique

In this study, a multistage-simple random sampling technique was employed to recruit 450 women who gave birth in the last 12 months for each group. Of the total rural Kebeles found in the district, seven Kebeles were selected using a simple random sampling method. Then the study participants grouped into PWC attendants and PWC non-attendants by reviewing their family matrix book found from each kebele’s health post based on pregnant women’s conference attending history. Women who didn’t attend the PWC during their last pregnancy considered as the PWC non-attendants and women who attended the PWC during their last pregnancy considered as the PWC attendants. The calculated sample size was, then, proportionally allocated to each Kebele’s and group’s based on the number delivery in the last 12 months. Next, a simple random sampling technique was applied to select the study participants for each group.

### Data collection

A pre-tested structured interviewer-administered questionnaire was used for data collection. The tool was prepared in English and translated to the local language, Amharic. Three female diploma midwives and one bachelor degree holder nurse were deployed as data collectors and supervisors respectively after receiving a one-day intensive training.

#### Operational definition

##### Well-prepared

A mother was considered as “well-prepared” if she made at least three from the four key types of birth preparedness and complication readiness practice during her last pregnancy (identified the skilled provider, saved money, planned health facility for delivery and identified the mode of transportation) before the onset of labor.

##### Knowledgeable on danger signs during pregnancy

A mother was considered as knowledgeable on danger signs during pregnancy if she spontaneously mentioned two or more of the three key danger signs these may occur during pregnancy (severe vaginal bleeding, swollen hands/face, and blurred vision).

##### Knowledgeable on danger signs during birth

A mother was considered as knowledgeable on danger signs during birth if she spontaneously mentioned two or more of the four key danger signs these may occur during childbirth (severe vaginal bleeding, prolonged labor (> 12 h), convulsion and placenta retained).

##### Knowledgeable on danger sign during postpartum

A mother was considered as knowledgeable on danger signs during postpartum if she spontaneously mentioned two or more of the three key danger signs these may occur during the postpartum period (severe vaginal bleeding, foul-smelling vaginal discharge, and high fever).

##### Data analysis

The collected data were entered into Epi Info V.7 and exported to SPSS Version 23 for analysis. Both descriptive and inferential statistics were done. In the analytical study, the first bivariable logistic regression analysis used to identify the independent effect of each on institutional delivery for each group. Variables having *P*-value ≤0.20 in the bivariable analysis were remained in the multivariable analysis to control the effect of confounders. Before doing independent logistic regression analysis among women who attended the PWC and didn’t attend the PWC independently, a significant difference between two independent groups was done since the study was comparative. Chi-square testing was done to see if there was any significant on prevalence of institutional delivery among mothers who attended and didn’t attend the PWC and a statistically significant difference was observed between the two groups (x^2^ = 17.98, df = 1, *p* = < 0.001), indicating that the factors associated with institutional delivery could be different among women attended and didn’t attend the PWC. Therefore, the analysis was conducted separately. Odds ratios (AOR) with their 95% CI were calculated to measure the strength of association, and *P*-value < 0.05 was considered as statistically significant.

## Results

### Socio-demographic characteristics of the respondents

Four hundred and fifty questionnaires were each distributed to both PWC attendant and PWC non-attendant respondents. However, 435 and 436 questionnaires were analyzed, yielding a response rate of 96.7 and 96.9%, respectively, women attended the PWC and didn’t attend the PWC. The mean age of women attended and didn’t attend the PWC was almost similar (30.9 ± 5.5 years VS 31.6 ± 5.1 years respectively). Almost all (93.1%) women were orthodox religion followers and the rest, 6.9% were Muslim religion followers. More than half (56.8%) of women who attended the PWC were living within 1 h of walking from the nearest health facility, whereas, 55% of women didn’t attend the PWC were living far away 1 h of walking. In both groups, the majority of women’s age group was 25-34 years (attended PMC: 75.8%, didn’t attend PMC: 60.9%) (Table 1).

**Table 1:** Socio-demographic characteristics of women who gave birth in the last 12 months, in the context of attending PWC, Northwest Ethiopia, 2017

Variable	Variable categories	PWC non-attendants (*n* = 436)	PWC attendants (*n* = 435)
		n (%)	n (%)
Age of women (years)	19–24	62 (14.2)	41 (9.4)
	25–34	252 (75.8)	265 (60.9)
	> = 35	122 (28)	129 (29.7)
Educational status	Unable to read and write	379 (86.9)	401 (92.2)
	Can read and write	0 (0.0)	2 (0.5)
	Grade 1-8	51 (11.7)	32 (7.4)
	Secondary and above	6 (1.4)	0 (0.0)
Occupation	Housewife	417 (95.6)	430 (98.9)
	Farmer	11 (2.5)	5 (1.1)
	Governmental employee	8 (1.8)	0 (0.0)
Marital status	Divorce	9 (2.1)	5 (1.1)
	Widowed	2 (0.5)	1 (0.2)
	Married	425 (97.5)	429 (98.6)
Family size	< 4	113 (25.9)	125 (28.7)
	> = 5	323 (74.1)	310 (71.3)
Husbands’ education	Unable to read and write	271 (67.8)	263 (61.3)
	Can read and write	102 (24.0)	118 (27.5)
	Grade 1-8	35 (8.24)	40 (9.3)
	Secondary and above	17 (4)	8 (1.9)
Husbands’ Occupation	Farmer	407 (95.8)	419 (95.6)
	Governmental employee	18 (4.24)	10 (2.3)

### Obstetric characteristics of respondents

The majority of women, 92.2% of women who attended the PWC and 78.0% of women who didn’t attend the PWC were had at least one ANC visit during their last pregnancy. About 1.4 and 3.4% of women who attended and didn’t attend the PWC had a history of stillbirth respectively (Table 2).

**Table 2:** Obstetric characteristics of women who gave birth in the last 12 months, in the context of attending PWC, Northwest Ethiopia, 2017

Previous parity	Null-Para	4 (0.9)	1 (0.2)
	1–2 births	86 (19.7)	91 (20.9)
	≥3 births	346 (79.40)	342 (78.9)
History of abortion	Yes	19 (4.4)	16 (3.7)
	No	417 (95.6)	419 (96.3)
History of stillbirth	Yes	15 (3.4)	6 (1.4)
	No	421 (96.6)	429 (98.6)
Ever birth at a health facility	Yes	171 (39.2)	217 (50.0)
	No	161 (59.4)	217 (50.0)
Had ANC follow up in last pregnancy	No	96 (22.0)	34 (15.0)
	Yes	340 (78.0)	401 (92.2)
Frequency of ANC visits	1-3 times	296 (87.1)	361 (90.0)
	4 and above times	44 (12.9)	40 (10.0)
Received BPCR counseling during ANC visit	No	55 (16.2)	33 (7.2)
	Yes	285 (83.8)	368 (91.8)
Discussed with partner/family about the place of delivery	no	195 (44.8)	119 (27.4)
	yes	241 (55.2)	316 (72.6)
Decision maker for the place of delivery during last birth	Self	149 (37.3)	123 (28.6)
	Husband	17 (4.3)	23 (5.3)
	Both (self+ husband)	233 (58.4)	284 (65.3)

### Awareness of obstetric danger signs

Almost all, 99.8% of women who attended the PWC and 95.4% of women who didn’t attend the PWC knew at least one key danger sign that may occur during pregnancy, childbirth or postpartum periods. Above three-fifth (61.8%) and two-fifth (40.4%) of women who attended and didn’t attend the PWC were mentioned sever vaginal bleeding as a danger sign during pregnancy, respectively (Table 3).

**Table 3:** Awareness of obstetric danger signs among women who gave birth in the last 12 months, in the context of attending PWC, Northwest Ethiopia, 2017

Variables	Categories	PWC non-attendants’ n (%)	PWC attendants’ n (%)
Danger signs			
During pregnancy	severe vaginal bleeding	176 (40.4)	269 (61.8)
	swollen of hands and face	151 (34.6)	214 (49.2)
	blurred vision	92 (21.1)	110 (25.3)
During childbirth	sever vaginal bleeding	118 (27.1)	182 (41.8)
	Prolonged labor (> 12 h)	129 (29.6)	174 (40)
	convulsion	35 (8.0)	68 (15.6)
	retained placenta	287 (67.4)	309 (71.2)
During postpartum	severe Vaginal bleeding	182 (42.7)	267 (61.5)
	Foul-smelling vaginal discharge	79 (18.5)	166 (38.2)
	high fever	11 (2.6)	53 (12.2)

### Knowledge of obstetric danger signs

**Fig. 1:**
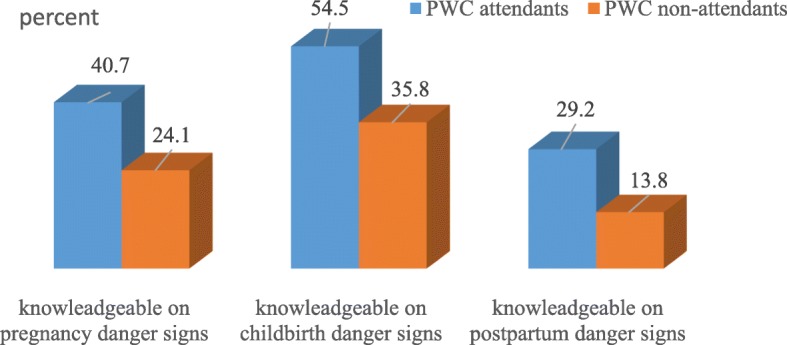
Knowledge of obstetric danger signs among women who gave birth in the last 12 months, in the context of attending PWC, Northwest Ethiopia, 2017

The proportion of women with adequate knowledge about danger signs that may occur during pregnancy was 40.7 and 24.1% for both women who attended the PWC and didn’t attend PWC, respectively (Fig. 1).

### Birth and its complication readiness practice during their last pregnancy

**Fig. 2:**
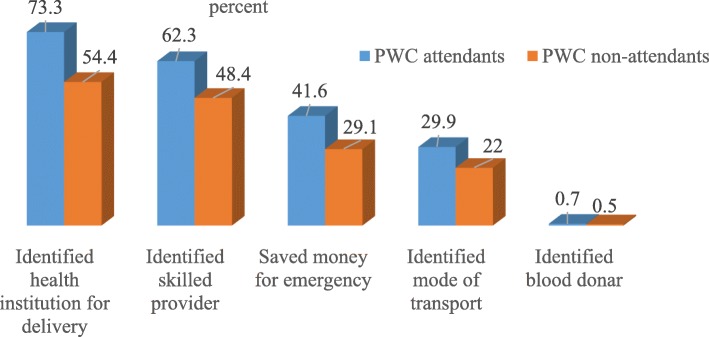
Birth preparedness and complication readiness practice among women who gave birth in the last 12 months, in the context of attending PWC, Northwest Ethiopia, 2017

Almost three-fourth (73.3%) of women who attended the PWC and half (54.4%) of women who didn’t attend the PWC, reported that they were planned health facility for delivery before the onset of labor (Fig. 2).

### Well-preparedness

**Fig. 3:**
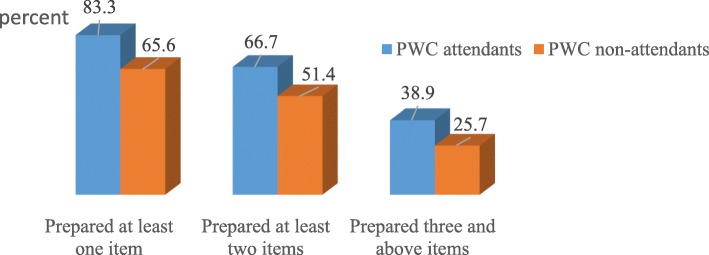
Well-preparedness during their last pregnancy among women who gave birth in the last 12 month, in the context of attending PWC, Northwest Ethiopia, 2017

Of the total, 38.9% of women who attended the PWC (95%CI: 33.8-43.7), and 25.7% of women who didn’t attend the PWC (95% CI: 22.2-29.4) were “well-prepared” for birth and its complication during their last pregnancy before the onset of labor (Fig. 3).

### The planned place for delivery and reasons

Almost one-fourth (26.7%) of women who attended the PWC and a half (45.6%) of women didn’t attend the PWC were planned to give birth at a home. Women feel more comfortable giving birth at home and need close attention from their families or relatives were frequently mentioned reasons why they planned to give birth at home in both groups (Table 4).

**Table 4:** The planned place for delivery and reasons to plan among women who gave birth in the last 12 months, in the context of attending PWC, Northwest Ethiopia, 2017

Variables	PWC non-attendants n1 (%)	PWC attendants n2 (%)
A planned place for the birth		
health institution	237 (54.4)	319 (73.3)
home	199 (45.6)	116 (26.7)
Why planned home for delivery (*n*1 = 199, *n*2 = 116)		
They feel more comfortable giving birth at home	121 (60.8)	65 (56.0)
Close attention from their families and relatives	98 (49.2)	54 (46.6)
Their usual practice	39 (19.6)	19 (16.4)
They didn’t like the service provided in health facilities	29 (14.6)	13 (11.2)
They had a bad experience giving birth at a health facility	4 (2.0)	2 (1.7)
Their families preference	16 (8.0)	3 (2.6)
The unwelcoming approach of health care workers	5 (2.5)	2 (1.7)

### Institutional delivery

The proportion of institutional delivery was 54.3% (*p* = 54.3, 95%CI: 49.9, 59.1) and 39.9% (*p* = 39.9, 95%CI: 35.3, 44.7%) among women who attended and didn’t attend the PWC, respectively. About 22.3% of women who attended the PWC and 13.5% of women who didn’t attend the PWC were had at least two PNC follow up during current delivery (Table 5).

**Table 5:** Institutional delivery among women who gave birth in the last 12 months, in the context of attending PWC, Northwest Ethiopia, 2017

Variables	PWC non-attendants n(%)	PWC attendants n(%)
Place of delivery		
At the health center	139 (31.9)	206 (47.4)
At hospital	35 (8.0)	30 (6.9)
Home	262 (60.1)	199 (45.7)
Why delivered at home after planned health institution (*n*1 = 88, *n*2 = 81)		
Labor was urgent to reach a health facility	28 (31.8)	32 (39.5)
Labor was coming at night and wait till dawn	32 (36.4)	47 (58.0)
Lack of transport	25 (28.4)	18 (22.2)
Lack of person look after home and care children	15 (17.0)	18 (22.1)
Family members prefer to deliver at home	17 (19.3)	8 (9.9)
The birth outcome of the current delivery		
Alive	428 (98.2)	432 (99.3)
Stillbirth	8 (1.8)	3 (0.7)
Postnatal care in the current delivery		
At least 1 visit	141 (32.3)	229 (52.6)
At least 2 visits	59 (13.5)	97 (22.3)
3 and above visits	10 (2.3)	23 (5.3)
No visit	295 (67.7)	206 (47.4)

### Factors associated with institutional delivery

On bi-variable, knowledge of danger signs in pregnancy, birth, and the postpartum period, traveling time to reach the nearest health facility, well prepared for birth and its complication, and discussion with partners/families about the place of birth were significantly associated with institutional delivery in both groups. On multivariable analysis, among women who attended the PWC, knowledge of danger signs that may occur during childbirth and the postpartum period, traveling time to reach the nearest health facility, well prepared for birth and its complication, and discussion with partners/families about the place of birth were significantly associated with institutional delivery. Whereas, among women who didn’t attend the PWC, knowledge of danger signs that may occur during pregnancy, traveling time to reach the nearest health facility and well prepared for birth and its complication were significantly associated with institutional delivery.

Among women who attended the PWC those who were knowledgeable on childbirth danger signs had 1.7 times higher odds of institutional delivery compared to women who were not knowledgeable on childbirth danger signs (AOR = 1.7, 95%CI: 1.2, 2.8). Whereas, the Odds of institutional delivery among women didn’t attend PWC those who had knowledge of pregnancy danger signs were 3.6 times higher compared with their counterparts (AOR = 3.6, 95%CI: 1.6, 8.1) (Table 6).

**Table 6:** Factors associated with institutional delivery among women who gave birth in the last 12 months, in the context of attending PWC, Northwest Ethiopia, 2017

Variables	PWC attendants				PWC non-attendants			
	I. delivery		COR	AOR	I. delivery		COR	AOR
	Yes	No			Yes	No		
Travel time to a nearby health facility								
< =1 h on foot	181	66	6.6 (4.3, 10.1)	4.4 (2.4, 8.1)*	131	65	9.2 (5.9, 14.4)	7.8 (4.4, 13.7*)
> 1 h on foot	55	133	1	1	43	65	1	1
Discussed with partner/family about the place of birth								
No	15	104	1	1	46	149	1	
Yes	221	95	16.1 (8.9, 29.0)	7.7 (3.6, 16.4)*	128	113	3.7 (2.4, 5.6)	======
Knowledge of at least two danger signs of pregnancy								
No	92	166	1		89	242	1	1
Yes	144	33	7.9 (5.0, 12.4)	========	85	20	11.6 (6.7, 19.9)	3.6 (1.6, 8.1)*
Knowledge of at least two danger signs of childbirth								
No	64	134	1	1	85	159	1	
Yes	172	65	5.5 (3.7, 8.4)	1.7 (1.2, 2.8)*	89	67	3.0 (2.0, 4.6)	======
Knowledge of at least two danger signs of postpartum								
No	113	195	1	1	115	261	1	
Yes	123	4	53.1 (19.1, 147.5)	14.0 (4.6, 40.0)*	59	1	133.9 (18.3, 978.3)	======
Well prepared								
No	77	189	1	1	88	236	1	1
Yes	159	10	39.0 (19.5, 77.9)	8.8 (3.9, 19.8)*	86	26	8.9 (5.4, 14.7)	3.3 (1.6,7.0)*

Key I. delivery = institutional delivery

*= statically significant associated at *p*-value< 0.05

## Discussion

According to this study, the proportion of institutional delivery among women who attended the PWC was 54.3%, higher compared with 39.9% among women who didn’t attend the PWC. Similarly, higher institutional delivery among women who involved in the community-based interventions was reported in the studies done in Burkina Faso (56% VS 36%), Eritrea (46.8% VS 51.2) and Guatemala (54.7% VS 31.2%) [15–17]. The reason might be, women who involved in the intervention expected that they were better informed about obstetric danger signs and birth preparedness, that enables women were better placed to make reasonable decisions [18]. Community-based behavioral changing interventions believed that increase institutional delivery, however, in the study done Kenya (28% VS 37%), Bangladesh (10.5% VS 12.5%), and India (22.5% VS 21.8%) institutional delivery among intervention groups similar or lower compared with not encompass in the interventions [19–21]. The reason might be a poor monitoring system of the interventions, and different socio-demographic characteristics of the participants.

The level of institutional delivery in both groups lower compared to studies done in Arba Minch (73.2%), Debre-Berhan (80.2%), and Ghana (63.3%) [22–24]. However, it was higher compared with the studies done in Dangla District (18.3%), and Banja District (15.7%) [25, 26]. The reason might be due to differences in socio-economic, and demographic characteristics of participants. Women who are educated, single, urban residents, and higher socioeconomic status are able to make wise decisions about their own health than their counterparts [18, 27–31].

This finding revealed that the awareness of obstetric danger signs in pregnancy, labor, and postpartum was higher among women who attended the PWC compared to those women who didn’t attend the PWC. This finding was in line with the studies done in Eritrea, and Bangladesh [16, 20]. On the other hand, acquiring of obstetric danger signs knowledge was not always consistency with related interventions. The studies conducted in Nepal and Bangladesh showed that obstetric danger signs knowledge of women who involved in the intervention were similar or lower compared to women those not participating in the interventions [32, 33]. The level of well-preparedness for birth and its complication practice was also significantly higher among women who attended the PWC, accounting 38.9% compared with 27.5% among women who didn’t attend the PWC. Similarly, in the studies done in Burkina Faso, Eretria, Nepal, and Tanzania the higher level of well-preparedness for birth and its complication was made among women who participated in the interventions [8, 16, 34, 35]. The reason might be women who involved in the intervention were better informed about birth preparedness and its complications.

The odds of institutional delivery among PWC attendant women who had knowledge of childbirth and postpartum danger signs were higher compared to their counterparts. On the other hand, among PWC non-attendant women who had knowledge of pregnancy danger signs were more likely to institutional delivery compared to their counterparts. Similarly, obstetric danger signs knowledge was positively associated with institutional delivery in the previous study done in Ethiopia, Pakistan, and Tanzania [18, 36, 37]. The possible explanation might be having knowledge of obstetric danger signs may influence women’s perceptions about their susceptibility to and seriousness of the complications. This might motivate women to give birth at health facilities [31].

In both groups, women who well-prepared for birth and its complication were more likely to institutional delivery. The reason might be women who were well prepared for birth and its complication might be knowledgeable about obstetric complications that may occur before, during and after birth; positively influence to give birth at a health facility.

Traveling time from the nearest health facility was significantly associated with institutional delivery in both women who attended and didn’t attend the PWC. In both groups, women who lived within 1 h of walking from the nearest health facility were more likely to institutional delivery compared to their counterparts. This finding was in line with other previous studies [18, 27, 28, 31]. The reason might be a lack of means of transportation to health facilities. Secondly, fear of financial cost for transport might be negatively influenced to decide institutional delivery.

The odds of institutional delivery among PWC attendant women who had a discussion with partners/families about the place of birth were higher compared with women who didn’t discuss. This may enable women to have autonomy in the choice of birthplace jointly. Women with the highest level of autonomy most likely to seek institutional delivery [36, 38–40]. In addition, this might create a better opportunity for families to involve in arranging transport, save money, and help mothers to choice a place of delivery.

## Conclusion

The proportion of institutional delivery was low in both groups compared to the national plan. However, it was higher among women who attended the conference. Similarly, women’s knowledge of obstetric danger signs and preparation for birth and its complication were higher among women who attended the conference compared with women who didn’t attend the conference. Pregnant women’s conference attendant women who had knowledge of childbirth and postpartum, and discussed with their partners/families about the place of delivery were more likely to institutional delivery. On the other hand, pregnant women’s conference non-attendant women who had knowledge about pregnancy danger signs were more likely to institutional delivery. In both groups, women who lived with one-hour of walking from the nearest health facility and well prepared for birth and its complication were more likely to institutional delivery. Therefore, strengthening women to attend pregnant women’s conference may improve institutional delivery by increasing women’s obstetric danger signs and birth preparedness knowledge. Furthermore, encouraging women to discuss with their families about the place of delivery should be strengthened. In addition, the government of Ethiopia better to strengthen the accessibility of health facilities for women.

## Data Availability

The data used to generate and or analyzed the current study are available from the corresponding author upon the request.

## Abbreviations

ANC: Antenatal care
AOR: Adjusted odds ratio
CI: Confidence interval
COR: Crude odds ratio
PWC: Pregnant women’s conference
